# Dr. James Andrew

**Published:** 1911-09-30

**Authors:** 


Dr. James Andrew, of Edinburgh, died last week in his sixty-eighth year. Dr. Andrew had a distinguished career in the University of Edinburgh, in which he graduated in 1866. After having studied abroad for some time, chiefly in Vienna and Berlin, he returned to his native city and was appointed to the Royal Hospital for Sick Children. He became Lecturer on the Diseases of Children at the University, and after being elected a Fellow of the Royal College of Physicians in 1870, as was his father, the late James Andrew, M.D. (Cantab.), he filled many important offices including that of Examiner in Medical Jurisprudence and Physician and Physician-Accoucheur to the Royal Dispensary. He was also a Fellow of the Obstetrical Society, Edinburgh.

